# Contributions of accreditation organizations in health services to the accreditation process: a bibliometric analysis

**DOI:** 10.1108/JHOM-11-2024-0488

**Published:** 2026-03-31

**Authors:** Fatih Orhan

**Affiliations:** Gülhane Vocational School of Health, University of Health Sciences, Ankara, Türkiye

**Keywords:** Bibliometric analysis, Thematic clustering, Accreditation bodies, Healthcare quality, Patient safety, Health services management

## Abstract

**Purpose:**

The purpose of this study is to investigate the concept of accreditation in terms of Accreditation Bodies in Health (ABIH) through a bibliometric analysis.

**Design/methodology/approach:**

Co-citation, co-occurrence and co-authorship analyses were performed on 465 academic publications selected from the Web of Science database focusing on health accreditation bodies. The analyses were conducted using R-based Bibliometrix software, Python and Microsoft Excel.

**Findings:**

The co-citation analysis identified seven themes: “Prevention of Clinical Errors and Patient Safety: Guidelines for Accreditation Bodies,” “Accreditation and Multidimensional Impacts on Healthcare Quality,” “Multidimensional Assessment of Hospital Quality in the United States: Standardization, Regulation, and Accreditation,” “Quality Assessment in Health Care: Theoretical Foundations and Personnel Management,” “The Complex Dynamics of Accreditation in Health Care: Analyzing Quality, Cost, and Performance,” “Quality and Performance Measurement in Health Care,” and “Accreditation and Pain Management.” Co-occurrence analyses revealed themes such as “Clinical Safety and Child Health,” “Beliefs and Ethical Issues,” “Patient Safety and System Errors,” “Guidelines and Risk Management,” “Quality and Performance Management,” and “Clinical Practice and Health Services.”

**Practical implications:**

The findings highlight the complexity of thematic categories and key issues in the literature on the contribution of accreditation bodies to the accreditation process in healthcare, which can inform policymakers and practitioners.

**Originality/value:**

This study provides a comprehensive bibliometric analysis of accreditation bodies in health, offering valuable insights into thematic clusters and contributing to the existing body of knowledge on healthcare accreditation.

## 1. Introduction

Quality and patient safety have been among the most explored healthcare topics for researchers, practitioners, and policymakers for many years. However, the effectiveness and sustainability of improvements in these areas are usually evaluated with the contributions of accreditation organizations (Roberts *et al*., 1987; Griffith *et al*., 2002; Chen *et al*., 2003; Greenfield and Braithwaite, 2008; El-Jardali *et al*., 2008). Accreditation bodies play a primary role in auditing and measuring the service quality of hospitals and healthcare organizations. The guidance and accreditation processes offered by these organizations effectively set quality standards for healthcare providers and improve patient safety. From a public health perspective, enhancing quality and patient safety in healthcare services contributes significantly to overall health outcomes in society. Accreditation processes help establish safer and more accessible healthcare services, playing a crucial role in protecting and improving public health.

In this context, accreditation bodies often establish effective methodologies and standards for the prevention of clinical errors, healthcare quality, and patient safety (Donaldson *et al*., 2000; Meinberg and Stern, 2003; Haynes *et al*., 2009; Donahue and Vanostenberg, 2000). However, in the existing literature, there is a lack of comprehensive studies evaluating the effectiveness of these organizations and analyzing the implementation results of the guidelines.

Studies examining quality and patient safety in healthcare in terms of accreditation have been the focus of academic and applied studies in recent years. In particular, prevention of clinical errors, (Donaldson *et al*., 2000; Meinberg and Stern, 2003; Haynes *et al*., 2009) accreditation processes, (Roberts *et al*., 1987; Griffith *et al*., 2002; Chen *et al*., 2003) hospital quality, (Williams *et al*., 2005; Brennan, 1998; Jha *et al*., 2005; McGlynn, 2003) theoretical foundations, (Donabedian, 1988a, b; Needleman *et al*., 2002) and the complex dynamics of accreditation (Greenfield and Braithwaite, 2008; El-Jardali *et al*., 2008). However, most of these studies either examined a specific theme in detail or focused on a specific management strategy.

This diversity poses challenges for health service managers and policymakers because, although the literature covers many themes and methods, it lacks a unified framework. Therefore, this study aims to fill the gap in this field by comprehensively analyzing the abovementioned themes and related literature.

The extensive and influential role of accreditation bodies on healthcare quality and patient safety has been widely discussed in the literature (Roberts *et al*., 1987; Griffith *et al*., 2002; Chen *et al*., 2003; Greenfield and Braithwaite, 2008; El-Jardali *et al*., 2008; Donaldson *et al*., 2000; Meinberg and Stern, 2003; Haynes *et al*., 2009; Donahue and Vanostenberg, 2000; Needleman *et al*., 2002). Firstly, accreditation organizations establish basic quality standards for healthcare services, enabling clinical practices to be evaluated and improved sustainably (Roberts *et al*., 1987). These standards are the first step of a continuous improvement process.

Another critical pillar of this process is periodic audits and evaluations. Through these audits, accreditation bodies continuously monitor and evaluate the practices of healthcare providers, thus supporting a continuous quality improvement process (Griffith *et al*., 2002; Chen *et al*., 2003). However, these audits are critical not only for quality but also for patient safety. For example, particular emphasis is placed on patient safety standards, preventing clinical errors, and improving patient outcomes (Haynes *et al*., 2009).

The impact of accreditation bodies is not limited to these audit mechanisms. They steer healthcare providers towards better practices by developing guidelines and protocols for specific diseases (Donaldson *et al*., 2000; Meinberg and Stern, 2003). They also positively impact organizational factors such as clinical alignment and staff management (Donahue and Vanostenberg, 2000; Williams *et al*., 2005; Brennan, 1998; Jha *et al*., 2005; McGlynn, 2003; Donabedian, 1988a, b; Needleman *et al*., 2002). This multifaceted approach can effectively improve health services' overall quality and efficiency (Greenfield and Braithwaite, 2008; El-Jardali *et al*., 2008).

Accreditation creates a bridge between policy and practice by facilitating the transition of health policies to the implementation phase. Moreover, the possibility of harmonization and cooperation between accreditation bodies in various countries facilitates the establishment of a global standard of quality and safety. Thus, accreditation bodies positively and multifacetedly impact healthcare quality and patient safety.

The key gap in the literature is that existing reviews—whether they focus on accreditation or on frameworks and models—do not examine the institutional contribution mechanisms of accreditation bodies as an independent unit within the accreditation process. Bibliometric and systematic studies on accreditation have generally advanced through topic-based co-word maps, maturity models, or outcome indicators (Waqas *et al*., 2019; Ke *et al*., 2016; Pagel and Hudetz, 2011). Actor-based contributions such as standard development, criteria architecture, evaluator–trainer capacity, feedback–corrective action cycles, and external benchmarking infrastructures often appear fragmented, difficult to trace within articles, or remain implicit. As a result, the contributions of accreditation bodies to the subject and process of accreditation remain largely invisible. Another overlooked issue is that comparative mapping across organizations does not distinguish between the concepts of “institutional contribution” and “overall quality/maturity.”

This study addresses that gap through an actor-centered and mechanism-based framework. By defining the unit of analysis as the organizational actor, it operationalizes contributions; standard and indicator production, measurement and evaluation tools, evaluator training, feedback, and benchmarking mechanisms are coded and compared at the institutional level. This design not only prevents scope creep but also renders the chain “institutional contribution → measurement infrastructure → reporting/learning” empirically traceable.

This study centers not merely on defining quality and patient safety in healthcare through standards, accreditation frameworks, or technological investments—as is commonly found in the literature—but rather on examining who enacts these regulatory structures in practice (from the perspective of accreditation bodies) and how they are operationalized in the field. This actor-centered positioning generates three original contributions.

First, it conceptualizes institutional assurance not as a static structure but as a dynamic implementation process, thereby uncovering the architecture of accountability. It reveals on whose shoulders safety and quality responsibilities rest, at which clinical or administrative tension points this responsibility becomes fragile, and where it effectively transforms into an ownerless domain.

Second, it locates the breakdown of the improvement cycle not through abstract policy discourse but through professional and practical micro-decisions—for instance, adherence to hand hygiene, complete transfer of information during patient handoffs, clarity in discharge instructions, or timely reporting of critical laboratory values. By doing so, it positions these everyday operational nodes as the true failure points of the system. This approach shifts the diagnosis of problems from a generalized abstraction such as “the hospital should improve quality” to the concrete question: “At which decision point, when a particular actor fails to act, does the cycle break?”—thus rendering the actionable leverage points visible and specific.

Third, it reframes the misalignment between regulatory expectations and field-level execution capacity not merely as a quantitative compliance gap (e.g. the percentage of standards met), but as a problem of institutional role architecture. As expectations grow around domains such as digital traceability, patient safety reporting, radiation protection, and clinical documentation integrity, the dispersion of these expectations across multiple actors—clinicians, quality managers, and IT infrastructures—transforms the issue from a technical performance deficit into one of organizational ownership. By making this role fragmentation visible, the study offers policymakers a directly applicable framework for addressing questions such as “Who should be responsible for which standard?” and “How should feedback loops be closed?”

In conclusion, the study complements and diverges from recent bibliometric/systematic reviews in three ways: it conceptualizes and measures actor-based contributions as an independent construct, integrates the bibliometric time series with the policy–standardization timeline to causalize periodization, and uncovers the mechanism layer in inter-organizational comparison, thereby enhancing the policy and practical value of the findings. These contributions provide quantitative and contextual evidence for the otherwise invisible institutional role of accreditation bodies in process architecture and systematically fill the actor–mechanism gap in the literature.

The aim of this study is to investigate the concept of accreditation in terms of accreditation bodies in health (ABIH) through a bibliometric analysis. Within this framework, the study addresses four main questions.

1. Which are the most prolific authors, most cited journals, institutions, countries, and trending topics studying the field of ABIH? Which are the most cited papers?
2. What are the most critical classical studies in terms of ABIH? What are the dynamics in the evolution of the intellectual structure of the field?
3. Can the conceptual structure of the ABIH field be identified?
4. Which authors stand out in the social, collaborative structure of the ABIH field?
5. Can the most common themes of the companies operating in the field of ABIH be tabulated?
6. How do the findings of a bibliometric analysis on accreditation bodies reframe quality governance along the axes of organizational accountability, micro-process bottlenecks, and technology-oriented role architecture?

The study has two main contributions to the literature. First, to the best of our knowledge, this is the first study to focus on accreditation from the perspective of ABIH. Second, through bibliometric and thematic analysis, we have provided valuable information on the themes of co-citation analysis and co-keyword analysis in the field of ABIH, thus contributing to identifying gaps to guide future research.

The article is divided into five sections. We start with the methodology in the second section, outlining the research technique. The results from the analysis are presented in Section 3, including performance analyses, bibliometric analyses, and the themes most studied by health accreditation bodies. Section 4 discusses the findings. In this section, we also present the limitations of the research and recommendations for future research. Finally, we conclude with a conclusion.

## 2. Method

**Figure 1:**
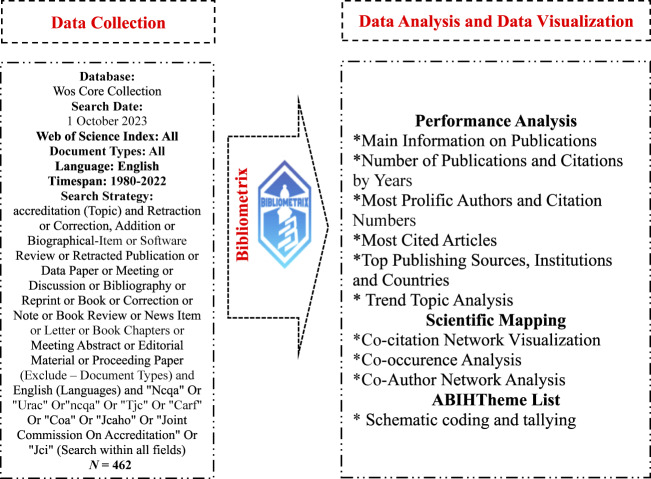
Flowchart of data collection, data analysis and data visualization

Bibliometric analysis is a useful method for examining large volumes of research data.It helps reveal relationships between publications, identify new research directions, and build a strong foundation for understanding the field. In particular, this type of analysis can help identify gaps in a field and develop new concepts. In academic circles, these analyses are often used to assess article and journal performance, identify collaboration networks, and identify trends in the field (Donthu *et al*., 2021). Web of Science (WoS) database was preferred in this study. Scholars often choose WoS for its advantages, such as detailed records, data sets suitable for bibliometric analysis, and indexing of prestigious publications. At the same time, this database is one of the most frequently used sources for bibliometric analysis (Kurutkan and Terzi, 2022; Zupic and Čater, 2015; Tengilimoğlu *et al*., 2024; Aydın and Orhan, 2025). WoS has an extensive collection of bibliographic lists, citation networks, and a number of full-text articles. Figure 1 shows the whole workflow of the analysis performed.

https://www.webofscience.com/wos/woscc/summary/763bd063-9fc2-4027-bf70-43ac9e0d1214-a88e9149/times-cited-descending/1.

During data selection, several filtering criteria were applied to refine the analysis scope.These filters ensured that only literature directly relevant to the study's objectives was included. First, only articles and review papers were included, while other types of publications such as conference proceedings, editorials, book chapters, and letters were excluded. This decision ensured the acquisition of data sets that more clearly reflect scientific impact and are methodologically comparable for bibliometric analyses (Donthu *et al*., 2021). As a language filter, only English publications were selected. This choice was made both to optimize the language processing capabilities of the analytical software employed and to enhance the comparability of the study within the international literature. The time span was set to [1980]–(2022) (with the first publication appearing in 1980), and the data published within this period in the Web of Science Core Collection were taken as the basis. This range was chosen to capture both the historical development of the field and its contemporary trends.

In this study, the search strategy was based solely on the official names and acronyms of accreditation organizations (“NCQA” OR “URAC” OR “TJC” OR “CARF” OR “COA” OR “JCAHO” OR “JOINT COMMISSION ON ACCREDITATION” OR “JCI”), and no word merging was applied. These terms are proper nouns and standardized institutional identifiers in the academic literature, without meaningful spelling variations. Unlike commonly used concepts (e.g. “health care” vs. “healthcare”), the institutional names in our query are not subject to orthographic variation that would require normalization. Therefore, applying word merging would not have yielded additional records or improved data quality. Preserving the original form of these terms ensured the methodological precision and reproducibility of the search strategy.

Since the search strategy targeted a closed set of standardized institutional names and acronyms for accreditation organizations, conceptual fragmentation was naturally minimized. Both full names and commonly used abbreviations were included in the query (e.g. “Joint Commission on Accreditation” and “JCAHO”; “National Committee for Quality Assurance” and “NCQA”), thereby capturing all common variations. As these are official identifiers, their usage in the literature is highly consistent, reducing the risk of fragmentation caused by synonyms or alternative spellings. For more generic terms such as “accreditation,” the search was restricted to contexts where these institutional identifiers also appeared, thereby further avoiding conceptual dispersion. This approach ensured the completeness and integrity of the dataset by capturing both abbreviated and full-name references.

On October 1, 2023, publications related to “accreditation” were searched in the Web of Science (WoS) database, and the search strategy was narrowed down to a targeted search for accreditation firms in parentheses (“NCQA” OR “URAC” OR “NCQA” OR “TJC” OR “CARF” OR “COA” OR “JCAHO” OR “JOINT COMMISSION ON ACCREDITATION” OR “JCI”). After applying various filtering strategies, a total of 462 publications were obtained. These data were analyzed using R-based, open-source Bibliometrix software (Karayel and Kurutkan, 2022; Aria and Cuccurullo, 2017; Bağiş *et al.*, 2023; Gülhan and Kurutkan, 2021). First, a performance analysis of these articles was conducted, including their basic statistics, authors, number of publications, journals, institutions, and countries. Then, a scientific map was created, including co-citation network, co-occurrence and co-author analysis. The analysis was performed without word merging. In the final stage, the abstracts and titles of the articles were reviewed to determine which themes each health accreditation organization was the subject of.

In performance and network analyses, the threshold values were determined based on the default parameters recommended by the relevant software (VOSviewer and Bibliometrix). This approach ensured the preservation of widely accepted standards in bibliometric analyses and guaranteed the comparability of results (van Eck and Waltman, 2010; Aria and Cuccurullo, 2017). For example, in performance analysis, the minimum citation threshold was set at five citations, which is the default value of VOSviewer, while for keyword co-occurrence analysis, the minimum frequency was set at three. Similarly, in author collaboration and institutional network analyses, the modularity and connection thresholds automatically defined by the software were adopted. This choice enhanced both the reproducibility of the analysis and its alignment with other studies in the literature.

In identifying thematic clusters, the Louvain and Walktrap clustering algorithms provided by VOSviewer and Bibliometrix were employed. The Louvain algorithm, based on modularity optimization, is capable of rapidly and efficiently detecting community structures in large-scale networks (Blondel *et al*., 2008). It is particularly preferred for identifying dense relationships among authors, citations, and keywords in bibliometric datasets. The Walktrap algorithm, by contrast, relies on a random walk approach to identify nodes with similar structures and high connectivity within the network (Pons and Latapy, 2005). This method is especially useful for detecting thematic proximities with high precision in smaller-scale or less modular sub-networks. Using both algorithms together made it possible to identify meaningful clusters at both macro (broad themes) and micro (sub-themes) levels. This approach overcame the limits of a single algorithm and provided a more comprehensive view of the thematic structures.

The choice of methods and tools directly aligned with the research questions: performance analysis (temporal trends, most prolific actors, sources) addressed RQ1; co-citation analysis identified the intellectual core and its evolution (RQ2); co-occurrence (co-word) analysis revealed the conceptual structure (RQ3); and co-authorship networks captured collaboration patterns (RQ4). The organization-theme list and schematic coding process systematically summarized the thematic foci across accreditation bodies, thereby informing RQ5. Bibliometrix, as an open-source, field-standard package supporting transparent and reproducible science mapping, was employed (Aria and Cuccurullo, 2017), while Python and Excel were used in preprocessing, tabulation, and visualization stages.

The selection of network-based techniques was consistent with best practices. Co-citation analysis remains the canonical approach for uncovering intellectual foundations and historical clustering, whereas co-word mapping captures contemporary conceptual proximities (Zupic and Čater, 2015; Donthu *et al*., 2021). To enable data-driven identification of cluster labels, network density and centrality measures (degree, betweenness, closeness, PageRank) were reported. To reduce the risk of over-normalization, institutional acronyms (e.g. JCAHO/TJC) were standardized for consistency, while author-provided keyword formats were preserved, and aggressive word merging was avoided. The inclusion thresholds and filtering rules, such as document types and query scope, were selected to balance data sparsity and interpretability.

These decisions were explained clearly in the methodology section and further discussed in the limitations section.

Taken together—database selection, time span, query design, and network techniques—the design was consistent, reproducible, and aligned with established guidelines, directly serving the five research questions (Aria and Cuccurullo, 2017; Donthu *et al*., 2021; Zupic and Čater, 2015).

## 3. Findings

The findings section consists of performance analyses, science mapping analyses, and analyses on which themes are the most relevant for accreditation bodies in health. The findings of this study were systematically linked to the research questions formulated in the introduction. Regarding the first research question, *“What are the bibliometric trends of publications in the field of accreditation in health services?*”, detailed performance indicators such as changes in the number of publications and citations over the years, the geographical distribution of publications, and the most prolific authors, institutions, and countries were presented. These trends reveal the historical trajectory of the field, highlight periods of acceleration, and illustrate the extent of international collaboration.

The second research question, *“Around which thematic clusters is the intellectual structure of this field shaped?”*, was addressed through thematic clusters generated by both co-citation analysis and co-keyword analysis. Each cluster was evaluated in terms of the topics it represents, the most influential publications within it, and the impact of these works on the field. This approach not only presented the cluster labels but also enabled interpretation of the relationships among clusters, conceptual proximities, and the flow of knowledge.

The third research question, *“What gaps and priority areas exist for future research?”*, was explored through the critical evaluation of the results derived from thematic cluster analysis. Alongside the literature that concentrates on specific themes (e.g. patient safety, quality indicators, cost-effectiveness analyses), less-studied or emerging themes (e.g. digital accreditation processes, AI-assisted quality assessment models) were identified. These findings were supported with concrete recommendations relevant to both academic research and policy development processes.

Through this comprehensive approach, the connections between research questions and findings were clearly demonstrated, thereby strengthening the interpretation of results and enhancing the study's contribution to the literature.

### 3.1 Performance analyses

#### 3.1.1 Bulgular (findings)

This study encompasses a total of 462 documents published between 1980 and 2022 on accreditation bodies. These documents were obtained from 296 different sources, indicating that the topic of accreditation has found representation in a wide range of journals. The average annual growth rate is 5.88%, demonstrating a sustainable increase in both interest and productivity in the field over time. The average age of the documents was calculated as 16.7 years, and each study received an average of 24.57 citations. In terms of author contributions, a total of 1,703 different authors have contributed to this body of literature. The rate of international collaboration stands at 7.143%, which is relatively low. An examination of document types revealed that 429 were research articles and 33 were review papers. This finding indicates that greater emphasis has been placed on empirical studies compared to conceptual analyses in review form. In addition, the number of *Keywords Plus* terms was 879, while the number of author keywords was 919. These figures demonstrate that both authors and indexers have focused on broad thematic domains.

**Figure 2:**
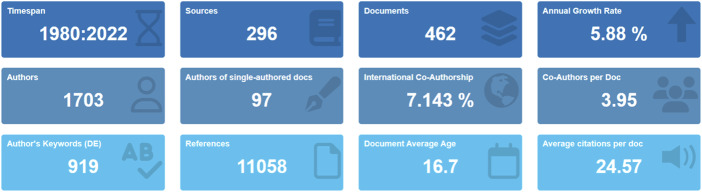
Main information

**Figure 3:**
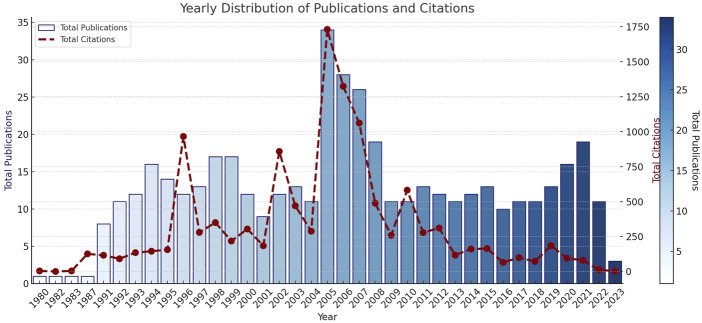
Number of publications and citations by year

When examining the changes in the number of publications and citations over the years (Figure 2), it is observed that between 1980 and 1990 only a very limited number of studies were published. Between 1991 and 2004, the number of publications remained relatively stable. In 2005, however, there was a notable increase in publications, marking a turning point in the field of accreditation. After 2005, the number of publications declined and followed a flat trajectory between 2010 and 2018. From 2019 onwards, a renewed increase in publications was observed. Citation counts increased in parallel with publications until 2010, after which they showed a gradual downward trend (see Figure. 3).

Within a dataset covering the years 1980–2022, the statement *“publications peaked in 2005”* is used to denote the turning point observed in the annual curve of publications and citations. The decision to highlight the post-2005 period rests on two considerations. First, the 2005 peak signifies the transition of the field into a maturity phase. Second, this same period marks the consolidation of performance measurement and safety standards. In the United States, the Hospital Quality Alliance catalyzed national public reporting of standardized process measures for acute myocardial infarction, heart failure, and pneumonia, publishing expanded indicators in April 2005. The Joint Commission's core measures had already been in effect since 2002, further reinforcing the link between accreditation and performance evaluation. Therefore, the focus on the post-2005 period is not only about the bibliometric “peak.” It also aims to capture the policy and standardization changes that transformed how quality and safety were measured and reported (Jha *et al*., 2005; Williams *et al*., 2005).

##### 3.1.1.1 Most published authors, institutions, journals, and countries

Among the most prolific authors, Loeb JM ranks first with six publications. He is followed by Hodge DR, Nadzam DM, and Williams SC, each with four publications. Other contributors with three publications include Burgess JL, Ehrmeyer SS, Fonarow GC, Gross PA, Kajo K, and Koss RG.

**Figure 4:**
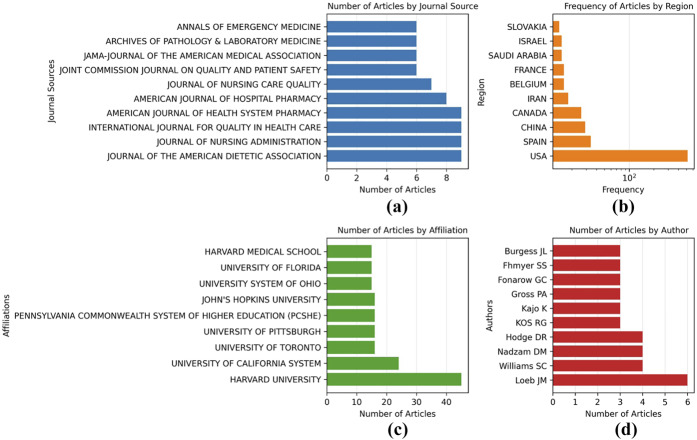
Authors, institutions, journals and countries with the most publications

In terms of the most productive institutions, Harvard University leads with 30 publications, followed by the University of California System with 24. Johns Hopkins University and the University of Toronto share third place, each with 16 publications. Harvard Medical School, Pennsylvania Commonwealth System of Higher Education, University of Florida, University of Pittsburgh, and the University System of Ohio are also notable contributors, each with 15 publications. It is noteworthy that some institutions (e.g. Harvard) appear under different names in the dataset (Figure 4).

With respect to journals, the American Journal of Health-System Pharmacy, International Journal for Quality in Health Care, Journal of Nursing Administration, and Journal of the American Dietetic Association top the list, each with nine publications. These are followed by the American Journal of Hospital Pharmacy with eight publications and the Journal of Nursing Care Quality with seven. Other leading sources include the Annals of Emergency Medicine, Archives of Pathology and Laboratory Medicine, JAMA, and the Joint Commission Journal on Quality and Patient Safety, each with six publications.

At the country level, the United States clearly dominates, followed by Spain, China, Canada, and Iran (Figure 4).

**Figure 5:**
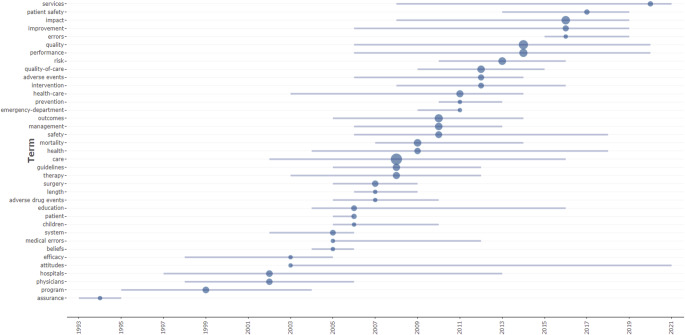
Trend topics

Trending Topics: According to the trend analysis, the most frequently used keyword is *“care,”* which appeared 45 times, particularly concentrated between 2002 and 2016. The term *“quality”* was used 26 times, and *“performance”* 18 times, both showing greater prominence during 2014–2020. The keyword *“health”* appeared over a broad period between 2004 and 2018, while *“patient safety”* was more common between 2013 and 2019, indicating a recent upward trend. The intensive use of the term *“services”* during 2020–2021 is also noteworthy. Other keywords—such as *“errors,” “patient safety,”* and *“services”*—emerge within narrower time intervals, reflecting period-specific research trends (Figure 5).

#### 3.1.2 Interpretations

The data show that research on accreditation has developed steadily over time. It is now well represented in the literature, both in scope and in depth. The average growth rate of 5.88% demonstrates that this field is not static but rather dynamic in nature. The fact that each publication received an average of 24.57 citations indicates that these studies have made not only a quantitative but also a qualitative impact on the literature. The average document age of 16.7 years suggests that much of this body of work dates back to the early 2000s, yet it has maintained its relevance.

The relatively low rate of international collaboration may be attributed to the country-specific structures of accreditation systems. Since national legislation, institutions, and regulatory mechanisms play a decisive role in the organization of healthcare services, research in this area is often conducted on a local scale. While this limits global comparisons, it also enables more in-depth analysis of local practices.

Institutions such as Harvard University, the University of California System, and Johns Hopkins University—among the most prolific contributors—demonstrate leadership in accreditation studies and occupy a decisive position in both research and practice. However, the occurrence of the same institution under different names in the dataset highlights the need for normalization (data cleaning). Such duplications can affect the accuracy of bibliometric analyses.

The journal analysis shows that accreditation studies are not confined to management- or quality-focused journals but span across multiple disciplines, including nursing, pharmacy, pathology, and emergency medicine. This multidisciplinary structure indicates that accreditation exerts an integrated influence across various components of healthcare systems. Nonetheless, a high number of publications in a journal does not directly indicate its academic authority in the field; factors such as impact factor, visibility within the field, and the quality of peer-review processes must also be taken into account.

The trend analysis shows how themes in the accreditation literature have evolved.

Earlier studies focused mainly on system organization, while recent work emphasizes quality, performance, and patient safety. This shift demonstrates the evolution of accreditation processes from a structural focus toward patient-centered quality approaches. Notably, the prominence of the term *“services”* in 2020–2021, under the impact of the COVID-19 pandemic, suggests that healthcare services were revisited in a more comprehensive and systemic manner. Low-frequency but recently concentrated concepts represent emerging yet noteworthy research themes.

Overall, this analysis reveals that the field of accreditation has developed both historically and in terms of content diversification. While it was more system-oriented in the past, it is now increasingly associated with contemporary issues such as patient safety, service quality, and performance measurement, thereby contributing to the ongoing transformation of healthcare systems.

### 3.2 Scientific mapping

In this section, co-citation and co-occurrence analyses were conducted. The name of each color was determined by using the prominent color clusters, and the intellectual structure, conceptual structure, and collaboration patterns of the articles studying the accreditation bodies in health services and related topics in the context of accreditation were determined.

#### 3.2.1 Co-citation analysis

**Figure 6:**
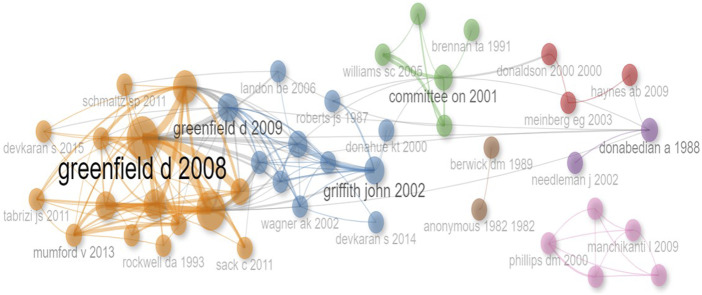
Co citation network analysis

Co-citation analysis allowed us to visualize the patterns of co-citation between research fields and determine the intensity and strength of the relationships between studies (Figure 6). After carefully reading the articles collected in each cluster, themes were named by the authors.

Theme 1. (Red color cluster in Figure 6) *Prevention of Clinical Errors and Patient Safety: Guidelines for Accreditation Bodies*: Donaldson *et al*. (2000), Meinberg and Stern (2003), and Haynes *et al*. (2009), who have made significant contributions in the field of patient safety and prevention of clinical errors, can provide guidance for accreditation bodies. The study of Donaldson *et al.* (2000) lays a foundation in this area and provides a systemic review of clinical errors. It draws a broad framework for accreditation bodies by emphasizing the importance of patient safety. Meinberg and Stern (2003) address errors in a specific area such as wrong-site surgery, and guide accreditation bodies on how to prevent such errors. Haynes *et al.* (2009) examine the effectiveness of surgical safety checklists from a global perspective and provide information on protocols that can reduce morbidity and mortality rates. Taken together, Donaldson *et al.* (2000) provide a general structure and framework, while Meinberg and Stern (2003) and Haynes *et al*. (2009) enrich this general framework with specific clinical scenarios and practical measures. In particular, while Donaldson *et al*. (2000) provide a basic structure in this area, Meinberg and Stern (2003) and Haynes *et al*. (2009) show that accreditation processes can be further refined by focusing on more specific clinical scenarios and measures.

Theme 2. (Blue color cluster in Figure 6) *The Multifaceted Impact of Accreditation Processes on Healthcare Quality*: This theme takes an in-depth look at accreditation processes and how these mechanisms impact the quality of healthcare services. The focus is on the implications of different assessment methodologies on patient outcomes and service quality. Related studies are discussed below within the framework of this theme:

In their study, Roberts *et al*. (1987) examined in detail the evolutionary history and organizational structure of the Joint Commission for Hospital Accreditation. This study sheds light on how accreditation organizations have evolved throughout history. Griffith *et al*. (2002) seek a balance between structural and outcome measures. Examining the correlation between Joint Commission and Medicare outcome scores, they discuss the impact of accreditation processes on healthcare quality. Chen *et al*. (2003) questioned the quality of care for acute myocardial infarction and the impact of JCAHO accreditation in this context. Donahue and Vanostenberg (2000) examine how Joint Commission International accreditation relates to different evaluation models. This study evaluates the synergy between international accreditation processes and local evaluation mechanisms. Greenfield and Braithwaite (2009) call for improving the evidence base of hospital accreditation processes. The study discusses current shortcomings and opportunities for improvement in terms of transparency and innovation of the process. Devkaran and O’Farrell (2014, 2015) analyze the impact of hospital accreditation processes on clinical documentation compliance. This study particularly highlights the importance of alignment between clinical practice and patient records. Landon *et al*. (2006) examine the quality of treatment of acute medical conditions in US hospitals and the possible impact of accreditation processes. Sack *et al*. (2010) investigate the impact of hospital accreditation on patient satisfaction in cardiology. The study points out that quality assessment should include the patient's perspective. This theme focuses on hospital accreditation and its impact on healthcare quality. Studies often address accreditation methodologies, patient outcomes, and clinical adherence.

Theme 3. (Green color cluster in Figure 6) *Multidimensional Assessment of Hospital Quality in the United States: Standardization, Regulation, and Accreditation*: This theme focuses on how hospital quality is measured, regulated, and improved in the United States. This theme is central to standardized measures, accreditation processes, and quality improvement strategies.

Williams *et al*. (2006) shed light on the evaluation criteria of accreditation bodies by analyzing standardized measures used in US hospitals. Brennan (1998) discusses how regulations play a role in the quality improvement process and evaluates the impact of accreditation. Jha *et al*. (2005) examine the quality of hospital care in the United States through the Hospital Quality Alliance program and determine the role of this program in accreditation processes. McGlynn (2003) examines the selection of common criteria used in quality assessments and provides a framework for understanding which criteria accrediting organizations prefer or may prefer. These studies address various aspects of hospital quality in the United States and explore the role of accreditation and regulatory processes. At the same time, they provide analytical frameworks for policymakers and accreditation bodies, guiding how hospital quality can be more effectively measured and improved.

Theme 4. (Purple color cluster in Figure 6) *Quality Assessment in Health Care: Theoretical Foundations and Personnel Management*: This theme offers a staff and process-oriented perspective on assessing quality in healthcare. Accreditation processes and quality assessments are the main elements of this theme. Donabedian (1988a, b) provides a comprehensive theoretical framework for quality assessment in healthcare. His work explores in depth the concepts underlying accreditation and quality assessments.

On the other hand, Needleman *et al*. (2002) discuss the effects of the number of nurses in a hospital on the quality of patient care. The study provides a notable perspective on how accreditation bodies address staffing arrangements. These two studies offer critical insights into assessing the quality of health care. While Donabedian (1988a, b) lays the theoretical foundations, Needleman and colleagues (2002) examine the impact of staffing arrangements on the quality of patient care in practice, namely in hospitals. These two studies provide critical insights into how accreditation processes evolve and what role these processes play in quality assessment. They also provide analytical frameworks for making accreditation and quality assessment more effective.

Theme 5 (Orange color cluster in Figure 6). *The Complex Dynamics of Accreditation in Healthcare: Analyzing Quality, Cost, and Performance*: This theme deals with various dimensions of healthcare accreditation. In particular, the articles comprehensively discuss how accreditation impacts quality, patient satisfaction, cost-effectiveness, and performance indicators.

Greenfield and Braithwaite (2008) examine the overall impact of accreditation processes on quality in a systematic framework, while El-Jardali *et al*. (2008) assess quality through nurses' perceptions of accreditation in Lebanon. These two studies provide a starting point for the impact of accreditation on quality. However, Brubakk *et al*. (2015) highlight the complexity of measuring the effects of accreditation, which can lead to methodological challenges. On the issue of cost, Mumford *et al*. (2013) discuss the possible effects of accreditation on quality in healthcare from a financial perspective, while Rockwell *et al*. (1993) specifically address the cost of accreditation for a hospital. In a Canadian context, Pomey *et al*. (2010) examine how accreditation processes incentivize healthcare organizations for organizational change. This is supported by Lam *et al*. (2018) and Schmaltz *et al*. (2011) both of which discuss the impact of accreditation on patient outcomes and performance indicators in US hospitals. The patient satisfaction dimension should not be ignored. Sack *et al*. (2011) investigate how hospital accreditation is related to patient satisfaction. On the other hand, Tabrizi *et al*. (2011) provide a comprehensive assessment of the advantages and disadvantages of healthcare accreditation models, which is also an essential part of the theme.

In conclusion, the articles we reviewed assess the impact of accreditation on healthcare from various angles and multiple perspectives. The theme can help us to comprehensively understand these complex interactions and the role of accreditation in the health sector.

Theme 6 (Brown color cluster in Figure 6) Quality and Performance Measurement in Healthcare: The main theme of the article “Berwick DM (1989)” in Cluster 6 can generally be considered as “Quality and Performance Measurement in Healthcare”. The article discusses EA Codman's influence on and struggle for quality and performance measurement in healthcare. Berwick's (1989) article discusses EA Codman's historical contributions to the improvement of health care quality. The author comments on Codman's struggle for quality measurement and improvement in health care. The article provides a historical perspective on the measurement and improvement of quality and performance in health care. Berwick's comments draw attention to the fact that Codman's innovative approaches in this field laid the foundation for the quality measurement and improvement strategies adopted by accreditation bodies today. In terms of the topic wer are studying, “how accreditation bodies examine accreditation”, this article provides an important commentary on the historical development of quality measurement and improvement strategies. It can also be a critical reference to better understand the historical roots of accreditation processes and how these processes can influence quality in healthcare.

Theme 7 (Pink color cluster in Figure 6) Title Proposal: “*Accreditation and Health Policy in Pain Management*”: Cluster 7 in the results of the co-citation analysis leans heavily on the theme of “Pain Management and Health Policy”. This thematic focus is reflected in a number of important studies. For example, Manchikanti *et al*. (2009) provide an algorithm for the clinical management of chronic spinal pain, providing a valuable framework for what quality and standards in pain management should be. Furthermore, Manchikanti and Singh (2008) provide a ten-year perspective on the medical and non-medical use of opioids, providing guidance for health policy making. The issue of why health care reform in the US needs radical change is addressed by Manchikanti *et al.* (2010) emphasizing how accreditation processes need to be changed. Prescription drug abuse and the political measures to be taken in this regard are discussed in detail by Manchikanti (2006). The impact of accreditation on the quality of pain management was discussed by Phillips (2000), who introduced the Joint Commission on Accreditation of Healthcare Organizations (JCAHO) standards in this field. In light of these articles, the most important study that can be directly related to the issue of “reviews of accreditation bodies” that our study focuses on is the study by Phillips. Phillips' study clearly demonstrates how accreditation standards can have an impact on pain management. Other articles provide clinical and policy guidance on how the accreditation process might take shape. Given this analysis, it would have been appropriate to rename the thematic focus to “Accreditation and Health Policy in Pain Management”.

The thematic clusters, visually distinguished by color, reveal distinct structural patterns within the literature, each reflecting a specific thematic focus. Every cluster represents a concentration of scholarly production around a particular topic. For instance, the cluster shown in orange includes highly cited central publications such as Greenfield and Braithwaite (2008) and Greenfield and Braithwaite (2009), indicating that this segment of the literature exhibits a dense and cohesive structure. The node sizes represent citation counts, and the relatively large size of the Greenfield and Braithwaite (2008) node suggests that this study has exerted a profound and guiding influence on the field.

The blue cluster is structured around Griffith *et al.* (2002) and shows notable interconnections with the orange cluster. This pattern implies thematic proximity and active knowledge exchange between the two clusters. In bibliometric terms, such boundary-spanning publications possess innovation potential capable of triggering the emergence of new research domains, as emphasized in Structural Variation Analysis (SVA) frameworks. Other clusters—depicted in green, red, purple, and pink—are less dense but point to thematically differentiated subfields. These may represent either narrowly defined sub-problems or newly emerging research directions.

The limited number of inter-cluster connections indicates that each cluster maintains a high degree of thematic integrity and internal coherence. Nevertheless, certain historical publications—such as Donabedian A (1988)—act as intellectual bridges linking different clusters and thus occupy a pivotal position in the historical development of the field. Such works generally constitute the “intellectual foundation” of the research domain.

Overall, this network structure reveals the knowledge architecture of the field, visualizing its core building blocks, flows of information, and potential paradigm shifts through central publications and clusters. This type of analysis is significant not only for identifying the current state of the literature but also for understanding its evolutionary trajectories and recognizing which studies have played a transformative role within the knowledge domain. Within the context of science mapping, this visualization serves as a powerful analytical tool that elucidates the organization of scientific knowledge, intellectual interactions, and the traces of scholarly transformation across the field.

#### 3.2.2 Co-occurrence analysis

**Figure 7:**
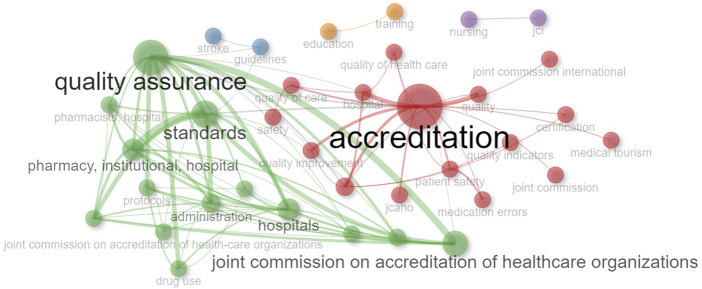
Co-Occurrence Network analysis. author's keyword

This co-occurrence analysis is based on parameters derived from articles in which accreditation organizations and the concept of accreditation are mentioned together (Figure 7). Each “node” represents a word or concept, and the relationships between these words were measured with different metrics such as “Betweenness,” “Closeness” and “PageRank.”

Some of the network analysis measures employed in this study may appear unfamiliar to readers without prior knowledge of bibliometric or scientometric research. To enhance clarity, we briefly define the main measures applied. PageRank, originally developed for Google's search algorithm (Brin and Page, 1998), evaluates the importance of a node (e.g. author, publication, keyword) not only by the number of links it receives but also by the significance of the nodes from which those links originate. Betweenness centrality quantitatively expresses the extent to which a node serves as a “bridge” connecting different parts of the network; nodes with high betweenness scores play a critical role in facilitating the flow of information (Freeman, 1977). Degree centrality measures the number of direct connections a node has, thereby indicating its immediate level of network activity (Borgatti *et al*., 2009). Closeness centrality reflects how quickly a node can reach all other nodes in the network via the shortest paths, thus capturing its potential efficiency in disseminating information (Sabidussi, 1966).

These measures were selected because they are widely recognized in bibliometric and co-word analyses for identifying influential nodes, structural positions, and thematic linkages (van Eck and Waltman, 2010; Donthu *et al*., 2021). Using them in combination allows for a more nuanced interpretation of thematic clusters and ensures that the discussion is grounded in measurable and replicable criteria.

The results of the co-occurrence analysis of this data are very interesting and show different aspects of accreditation and related Topics (Figure 7). Each “cluster” can be thematically named as follows:

1. Cluster 1 (Red color in Figure 7): Accreditation and Quality Standards:

This cluster contains keywords strongly linked to accreditation and quality standards.

They include terms such as accreditation, patient safety, healthcare quality, hospital management, certification, and medical tourism. Accreditation serves as an assurance that patients are safe and that healthcare services are of high quality. Concepts such as quality and patient safety are directly linked to accreditation. The existence of medical tourism in this cluster also suggests that accreditation is an important factor for medical tourists in determining their destination to fulfill their healthcare needs.

- Cluster 2 (Blue color in Figure 7): Medical Protocols and Guidelines:

The keywords, such as guidelines and stroke in this cluster, suggest the essential role of medical protocols and guidelines during accreditation. The accreditation process often requires healthcare providers to implement specific guidelines and protocols.

- Cluster 3: Healthcare Organizations and Accreditation:

This cluster includes keywords closely related to healthcare organizations.

These terms cover topics such as quality assurance, accreditation standards, hospital management, pharmacy services, documentation, compliance, and prescribing practices. Accreditation promotes harmonization and quality assurance in healthcare organizations, covering healthcare management processes such as standards and certification, drug utilization, and prescribing.

- Cluster 4: Nursing:

The keywords such as jci and nursing suggest that nursing is integral to the accreditation process. For health professions such as nursing, accreditation can guarantee the quality of education.

- Cluster 5: Education and Capacity Building:

The keywords such as education and training suggest that education and capacity-building through training are necessary for generating the healthcare workforce. Accreditation promotes continuous education and capacity building of health professionals.

When all clusters are analyzed together, a clear overall pattern emerges. Indicators such as Betweenness, Closeness, and PageRank show that the terms *“accreditation”* and *“quality assurance”* occupy central positions. This centrality suggests that these concepts are strongly related to other accreditation elements. For example, the high “Betweenness” value of the word “accreditation” indicates that it acts as a “bridge” between different concepts, while the high “PageRank” value of “quality assurance” indicates that it has more importance than other concepts.

General Evaluation on Explaining Thematic Clusters with Concrete Data: Each node represents a keyword, while the connections (edges) between nodes indicate that these terms co-occur within the same documents. Colors represent clusters; node sizes reflect either the frequency of co-occurrence or centrality measures (e.g. betweenness centrality).

At the center of the network, the large red node “accreditation” emerges as a highly frequent term that is directly associated with many other concepts in the literature. The size of this node indicates both its frequency of use and its central role in the knowledge domain. Terms within the same cluster, such as “quality of care,” “certification,” “patient safety,” and “quality indicators,” demonstrate that accreditation is directly linked to service quality, highlighting that patient safety and quality indicators are the primary themes of research in this area.

On the other hand, the green cluster is represented by terms such as “quality assurance,” “standards,” and “joint commission on accreditation of healthcare organizations.” This cluster reflects administrative and institutional frameworks aimed at ensuring quality in healthcare services. Specifically, terms such as “standards,” “protocols,” “administration,” and “drug use” represent literature focused on quality control at the operational level. The density of internal connections within this cluster suggests that these terms frequently co-occur and that this subfield has a well-defined structure.

Notably, significant connections exist between the two main clusters, showing that quality assurance and accreditation processes are addressed together in the knowledge domain and evolve interactively. For instance, nodes such as “hospitals” and “joint commission” act as bridges between the clusters, maintaining thematic coherence.

Smaller clusters represented in purple, blue, and orange reflect more specific or emerging subthemes. For example, the purple cluster, containing nodes such as “nursing” and “jci,” may represent accreditation processes within the nursing context. The orange cluster, with terms such as “education” and “training,” highlights the link between accreditation, organizational learning, and professional development.

Overall, this network structure successfully illustrates the multidimensional nature of accreditation in healthcare services and the main conceptual focuses in the literature. The two major themes shaped around “accreditation” and “quality assurance” show how quality management in healthcare is structured at both policy and operational levels, while the emerging subthemes trace the evolutionary directions of the field. In this sense, the network not only reflects the existing body of knowledge but also holds high value for anticipating future research orientations.

#### 3.2.3 Co-author analysis

**Figure 8:**
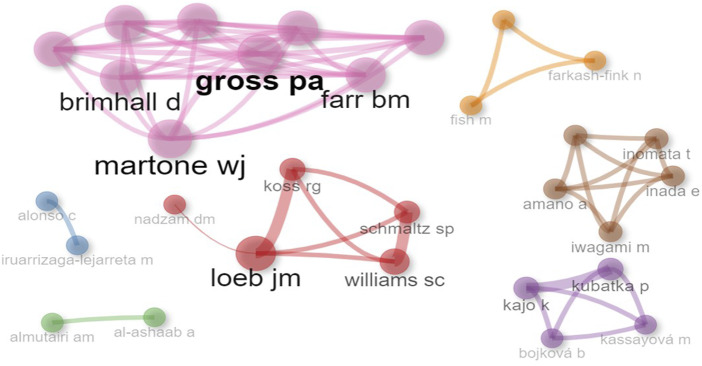
Co-author network analysis

Clustering: In Figure 8, we see that there are seven different clusters in total. The authors in Cluster 1 (Loeb JM, Nadzam DM, Williams SC, Koss RG, Schmaltz SP) focused on performance measures of accreditation and quality of care (Williams *et al*., 2006; Nadzam and Loeb, 1998). Martone and Gross PA collaborated the most on antimicrobial resistance and infection control mechanisms in the context of accreditation requirements (Goldmann *et al*., 1996; Scheckler *et al*., 1998). The co-authorship group of Inomata, Amano, and Iwagami have examined the impact of accreditation on surgical processes and the organization of operating rooms. Other clusters were not analyzed as they hosted few collaborations.

Betweenness: “Loeb JM” has a high betweenness score (3), indicating that it acts as a “bridge” between other authors. Other authors usually have a mediation score of 0, indicating they do not occupy a central position in the network. Closeness: Authors with a closeness score of 1 (e.g. Alonso C, Iruarrizaga-Lejarreta M) may have a central position in the network. This indicates that these authors can easily collaborate with others. PageRank: “Loeb JM” is the author with the highest PageRank (0.045). This indicates its importance in the co-author network. Comparisons: Comparing Cluster 1 and Cluster 4, the authors of Cluster 1 have higher PageRank values, while Cluster 4 has generally higher proximity scores. There does not seem to be a significant correlation between proximity and PageRank. For example, “Alonso C” scores 1 in proximity, while it has a low value (0.033) in PageRank. In conclusion, this figure shows the relationship between the authors in the co-author network and their importance in the network with different metrics. In particular, “Loeb JM” stands out as a versatile author. The other authors seem to interact mostly within their own clusters.

#### 3.2.4 Themes that accreditation bodies study the most

This list outlines the main focus areas of accreditation bodies.

Each organization has its own standards and processes, but most emphasize patient safety, staff training, quality improvement, satisfaction, and risk management. However, each institution has its own focal points that may change over time. While these organizations have mutually similar themes, each focuses more on certain topics according to its area of expertise. For example, JCI focuses more on international standards and quality improvement, while NCQA offers more specific standards for healthcare providers and insurance companies in the United States. JCAHO accredits hospitals and long-term care facilities, while NCQA evaluates health plans and physician-to-practitioner services. URAC focuses on the effective and efficient delivery of health services, while ISO focuses more on quality management systems. AAHRPP usually focuses on clinical research and human rights protection. When making a comparison between accreditation bodies, it is important to take such differences into account.

Accreditation bodies operating in the health sector generally establish certain standards and criteria to improve the quality and safety of health services. Different accreditation bodies may focus on different topics and areas. Below are a few important accreditation bodies and the topics they usually focus on:

1. Joint Commission International (JCI): This organization covers a wide range of topics such as patient safety, healthcare quality, hospital management and staff training.
2. Accreditation Association for Ambulatory Health Care (AAAHC): Establishes standards for ambulatory services, surgical centers and other healthcare providers. The main themes it focuses on are patient safety and quality care.
3. Commission on Accreditation of Rehabilitation Facilities (CARF): Focuses on issues such as rehabilitation and chronic disease management. It also covers elderly care and services for people with disabilities.
4. National Committee for Quality Assurance (NCQA): Conducts quality assessments for health plans, health care providers and other organizations. Focuses on topics such as chronic disease management, patient-centered care and digital health records.
5. College of American Pathologists (CAP): Provides accreditation for pathology laboratories. Main focus areas are laboratory safety, test quality and patient safety.
6. ISO 15189: An accreditation standard for medical laboratories. It specifically covers laboratory safety, quality control and patient safety.

##### 3.2.4.1 Healthcare accreditation bodies

1. JCAHO (277 publications)
2. JCI (42 publications)
3. TJC (12 publications)
4. NCQA (15 publications)
5. URAC (5 publications)
6. ISO (8 publications)
7. AAAHC (4 publications)
8. CARF (8 publications) is represented in the database. The contribution of each institution will now be briefly clarified.

The Joint Commission on Accreditation of Healthcare Organizations (JCAHO) has been evaluating and accrediting healthcare organizations for many years (see Table 1). Throughout the accreditation process, JCAHO addresses many different themes. In this study, we examined the themes that JCAHO focuses on the most under two main pillars: “Most Cited Studies” and “Recent Studies” (Table 2). Accreditation requires a comprehensive and multidisciplinary approach to ensure patients receive the best care. However, there may not be consensus on which aspects of this field should be focused on more. In two main thematic groups, the most cited studies, on the one hand, and recent studies, on the other, show which areas of accreditation have seen significant developments.

**Table 1:** Accreditation Bodies, programmes and site names

Name	Full name	Healthcare settings	Website
NCQA	National Committee for Quality Assurance	Health plans, providers, and medical groups	www.ncqa.org
URAC	Utilization Review Accreditation Commission	Health plans, pharmacies, and provider organizations	www.urac.org
TJC	The Joint Commission	Hospitals and other healthcare organizations	www.jointcommission.org
CARF	Commission on Accreditation of Rehabilitation Facilities	Rehabilitation facilities, behavioral health organizations, and employment services	www.carf.org
COA	Council on Accreditation	Social service and behavioral health organizations	www.coanet.org
JCAHO	Joint Commission on Accreditation of Healthcare Organizations	Hospitals and health care organizations	This organization is now known as TJC.*
JCI	Joint Commission International	Healthcare organizations outside the USA	www.jointcommissioninternational.org

**Note(s):** *The abbreviation JCAHO has been known as TJC since 2007, thus has the same website as TJC

**Table 2:** Themes that accreditation bodies study the most

JCAHO (277 publications)		Joint commission international (42 publications)
Most cited studies	Recent studies	JCI publications
Control of Antimicrobial Resistant Microorganisms	Quality Indicators and Performance Measurement	Hospital Accreditation and Quality Measures
Pain Management in the Emergency Department	Hospital Management and Leadership	Patient Safety and Medication Management
Medication Errors	Emergency Preparedness	Clinical Nutrition Services
Opioid Use	Hospital Physical Conditions and Safety	Operating Room Processes and Performance
Quality in US Hospitals with Standardized Measures	Patient Safety and Risk Management	Medical Education and Quality Management
Hospital Quality for Acute Myocardial Infarction	Pain Management	International Patient Safety Goals
Patient Safety Activity Taxonomy	Medicines Management and Safety	Sterilization and Management of Environment and Equipment
Operating Room Teamwork	Infection Control and Epidemiology	Hospital Staff Training and Event
Compliance with Heart Failure Quality Indicators	Operational and Clinical Standards	Patient and Family Centered Care
Treatment Based on Numerical Pain Assessments	Teamwork and Communication	Emergency Service Accreditation
Troubled Doctors and System Level Solutions	Information Technology and Electronic Health Record	Radiation Safety and Management
Prevalence of Incorrect Level Surgery	Documentation and Reporting	Microbiology Laboratory Accreditation
Procedural Sedation and Risk of Pulmonary Aspiration	Training and Trainer Evaluations	Anesthesia and Perioperative Care
Spiritual Assessment Template	Patient Rights and Ethics	Hospital Infection Control Programs
Changes in Methadone Treatment Practices	Patient Assessment and Care Planning	Staff Productivity and Motivation
Cancer Pain Management	Procedural Standards and Practices	Promotion of Patient Rights
Infection Control and Hospital Infrastructure Requirements	Laboratory and Diagnostic Services	Quality Indicators and Performance Measurement
Eradication of “Never Events”	Chronic Disease Management	Factors Affecting Re-hospitalization of Patients
Patient Handover Systems	Psychiatric and Mental Health Services	Accreditation of Primary Health Care Services
Laboratory Critical Value Reporting	Anesthesia and Surgery Management	The Role of the Mortality Committee in Quality and Safety
Discharge Instructions Affecting Re-hospitalization of Patients	Child and Adolescent Health	Service Quality Assessment in Radiology Departments
Quality of Care for Acute Medical Conditions	Palliative and End of Life Care	Audit according to Accreditation Standards for Emergencies
Continuous Process Improvement and Nutrition Care	Cardiovascular Services	Patient and Family Centered Care: A Primary Health Care Perspective
Procedural Sedation and Analgesia Applications	Women's Health and Maternity Services	Radiodermatitis Management and Radiation Safety
Radiation Dose Estimation and Comparison	Oncology Services	Human Resources and Personnel Management
Coding of Birth Complications	Telemedicine and Remote Health Services	Managerial and Organizational Strategies
Quality of Perioperative Care	Bioterrorism and Emergency Management	Analytic Hierarchy Process for Improving Service Quality
Sociotechnical Probabilistic Risk Modeling	Nutrition and Dietetics Services	Cooperation in Cultural Exchange and Accreditation Process
Hand Washing Compliance Rates	Physical Therapy and Rehabilitation	Patient-Centered Care Practices for Patients with Subarachnoid Hemorrhage
Smoking Bans in American Hospitals	Medical Devices and Equipment Management	Use of Referral Laboratories and Accreditation Standards
Spiritual Assessment and Intervention	Bed and Out of Bed Patient Management	Total Quality Management to Improve the Quality of Health Services
Pain Management After Anterior Cruciate Ligament Repair	Employee Health and Safety	Quality and Safety of Hospital Infection Control Programs
Bioterrorism Preparedness	Drug Form Management and Stock Control	Productivity and Motivation of Laboratory and Blood Bank Employees
Bringing Quality Improvement to Intensive Care Units	Multidisciplinary Team Coordination	Hospital Accreditation Processes in Saudi Arabia
Order Sets for Clinical Decision Support	Health Literacy and Patient Education	Impact of JCI Accreditation Process on Employees of Four Sectors
Acute Myocardial Infarction Treatment Processes and Outcomes	Patient Satisfaction and Feedback	Applicability of Accreditation Standards in Primary Health Care Centers
Pain Assessment for Older Adults with Hip Fracture	Clinical Research and Ethics	
ECG Examination and Emergency Service Applications	Radiation Safety	
Restraint and Sedation Practices in the Emergency Department	Organ and Tissue Donation and Transplantation	
Development of Drug Utilization Indicators	Medical and Legal Transactions	

##### 3.2.4.2 Common points

*Pain management* is among both the most cited and recent studies. This particular focus indicates that pain management is a critical issue and directly affects the quality of life for patients. Medication Management and Safety: The correct use and management of medicines is critical to patients' health. Medication errors and medication management are also seen in both themes. Patient Safety and Risk Management is among the most cited and recent studies. This shows the importance that healthcare organizations attach to this topic.

##### 3.2.4.3 Differences

Recent studies include more technology-oriented topics such as information technology and electronic health records. The most cited studies focus more on clinical practices, such as procedural sedation and the risk of pulmonary aspiration. Recent studies focus more on organizational and administrative issues such as hospital management and leadership.

These two sets of themes show that accreditation is not limited to medical practices but encompasses organizational, technological, and legal aspects. Clinical and administrative aspects are elements that need to be considered for the effective functioning of healthcare organizations. Understanding these themes can guide health managers, clinical staff and policy makers on which areas to focus more on.

Contributions of Other Organizations (Table 3): Different organizations have different focuses for health accreditation and quality improvement. Here are key themes from publications by organizations such as URAC, NCQA, ISO, and CARF:

**Table 3:** Contributions of other organizations

URAC 5 publications	NCQA 15 publications
Public Agenda Setting for Online Health Searches	Cholesterol Management for Coronary Heart Disease
Quality Improvement and Accreditation Organizations	Predictors of Health Plan Accreditation
Performance of Assured Hospitals on National Quality Care Process Measures	Accreditation Bodies and Quality Improvement
Incentives for Medicare Prescription Drug Plans to Consider Long-Term Outcomes and Cost	Championship Management for Healthcare Organizations
Feasibility of a New Specialty Pharmacy Reported Measure Defining Treatment Completion for Hepatitis C	Adolescent Vaccinations
*ISO 8 publications*	Asthma Quality Improvement Projects
JCI Accreditation and the Four Assessment Models	Quality Measurement for Health Systems
The Evolution of External Quality Assessment from the Joint Commission on Healthcare Accreditation	NCQA Efforts for Value Seeking
The Quest for a Quality Blood Banking Program in the New Millennium	Performance Indicator as Discharge Status
Performance of Assured Hospitals on National Quality Care Process Measures	Assured Hospital Performance
ISO System 9,000 or Assessment of the Quality of Medical Care	NCQA PCMH Recognition in Family Medicine Residency Practices
Using the SLIPTA Checklist to Assess Laboratory Readiness for JCI Accreditation	3 Different Performance of the Medical Home Recognition Program
ISO 15189 Accreditation Experience in Turkish University Hospital Microbiology Laboratory	Approval of Services Supporting Compliance with Health Standards
ISO 15189-Accredited Laboratories, Referral Laboratories and JCI Hospital Accreditation Standard	Factors Affecting HMOs' NCQA Accreditation Decisions
*CARF 8 publications*	Quality Indicators Related to NCQA Accreditation Level
Age Discrimination and Rehabilitation Professionals	*AAAHC 4 publications*
Predicting Adolescent Substance Abuse Treatment Outcomes	Establishment of Office Based Surgery Program in Gynecologist's Office
National Quality Measures in Assured Hospitals	3 Examining the Different Performance of a Medical Home Recognition Program
Accreditation of Results-Oriented Information Systems	Preparation for Accreditation
Implementation of the new CARF Wellness Standards	Office Accreditation in Dermatology
Criteria for Short-Term Rehabilitation in Nursing Homes in the USA	
Outcome-Based Standards for Substance Abuse Rehabilitation Programs	
Developing a Physical Restraint Policy for Acute Care	

##### 3.2.4.4 Common points

Most organizations have published on accreditation processes and quality improvement strategies. For example, topics such as “Quality Improvement and Accreditation Bodies” and “Predictors of Health Plan Accreditation” affect many organizations. Organizations often included hospitals and health systems. For example, topics such as “Performance of Assured Hospitals on National Quality Care Process Measures” are covered by both URAC and CARF. Themes such as “Championship Management for Healthcare Organizations” or “NCQA PCMH Recognition in Family Medicine Residency Practices” offer a common focus on quality indicators and performance measurement.

##### 3.2.4.5 Differences

While NCQA addresses more specific diseases and conditions, such as “Cholesterol Management for Coronary Heart Disease” or “Adolescent Immunizations”, URAC often focuses on broader themes, such as “Setting the Public Agenda for Online Health Searches”. ISO generally focuses on technology and standardization (“ISO 15189-Accredited Laboratories”), while NCQA focuses more on healthcare management and quality indicators (“Factors Influencing NCQA Accreditation Decisions for HMOs”). CARF focuses more on rehabilitation and quality of life issues. For example, themes such as “Age Discrimination and Rehabilitation Professionals” or “Outcome-Based Standards for Substance Abuse Rehabilitation Programs” are prominent for this organization.

These different organizations and publication themes show that the health sector is multifaceted and complex. However, accreditation and quality improvement are a common focus for all organizations, and each addresses different aspects in their respective areas of expertise. Therefore, health managers and policymakers must consider these differences and commonalities to develop more effective and comprehensive strategies.

These organizations may focus on specific issues, but they usually focus on many issues under the main theme of patient safety and healthcare quality. When making inter-agency comparisons, it is imperative to consider which types of healthcare organizations they target and which issues they emphasize more. This type of analysis can help us better understand how accreditation bodies play a role in various health and governance issues.

A chronological comparison of the development and focus areas of major accreditation bodies, including ISO, TJC (JCAHO), CARF, NCQA, and URAC, is presented in Table 4.

**Table 4:** Chronological comparison table

Year/Period	ISO	TJC (JCAHO)	CARF	NCQA	URAC
1947–1950	ISO founded; foundation of quality management standards established	–	–	–	–
1951	–	JCAH (later TJC) was founded; initiated hospital accreditation focusing on quality and safety	–	–	–
1966–1969	ISO 9000 series began; quality management principles were developed	TJC published the first national patient safety standards	CARF founded (1966); began accreditation in rehabilitation services	–	–
1970s	ISO quality management spread globally	TJC expanded patient safety guidelines	CARF strengthened standardization in mental health and elderly care	–	–
1980s	ISO 9001 quality management standard published	TJC began initiatives focused on performance measurement	CARF engaged in international accreditation activities	NCQA founded (1979) and began accreditation activities in the late 1980s	URAC founded (preparatory period before 1990)
1990s	ISO 9001 revisions began to adapt to the healthcare sector	TJC developed Clinical Performance Measurement Sets	CARF included disability, independent living, and children's programs in accreditation	NCQA launched the HEDIS measurement set (1991); turning point in quality measurement for U.S. health plans	URAC founded (1990); focused on health plans, e-health, and telehealth accreditations
2000s	ISO 15189 (2003) published; became critical for laboratory accreditation	TJC introduced “National Patient Safety Goals” (2002), making patient safety systematic	CARF expanded accreditation to addiction treatment and child services	NCQA developed accreditation for chronic disease management and patient-centered medical homes (PCMHs)	URAC expanded accreditation to health insurance and e-health
2010s	ISO 9001:2015 revision strengthened risk-based approach	TJC made digital reporting and data-based improvements	CARF expanded international partnerships	NCQA introduced care-based and digital measurement sets	URAC took a pioneering role in digital health and telehealth accreditation
2020–2025	ISO standards focused on sustainability and digitalization	TJC adapted accreditation processes after COVID-19	CARF developed remote access accreditation	NCQA focused on digital quality measurement (Digital HEDIS)	URAC provided pioneering accreditations in telehealth, medication management, and digital health services

The chronological table shows that the five major organizations in healthcare accreditation contributed at different times and in different contexts. Among them, ISO appeared first, setting a global framework for quality management standards. This approach focused on quality management processes rather than direct healthcare accreditation. Beginning in the 1950s, TJC institutionalized patient safety and clinical quality standards, creating an accreditation model tailored specifically to healthcare institutions. CARF specialized in rehabilitation, mental health, and services for individuals with disabilities, thus covering a narrower but highly critical domain. In the 1990s, NCQA made a turning point with the HEDIS measurement set, enabling the assessment of health plan performance, and played a significant role in chronic disease management and patient-centered care models. URAC, from the 1990s onward, pioneered innovative accreditation models in digital health, e-health, and telehealth, becoming the institution most rapidly adapting to technological transformation.

Overall, ISO's global quality management standards provided an overarching framework, while TJC established the first comprehensive accreditation model centered on patient safety and quality. CARF contributed to the diversification of healthcare accreditation by focusing on specialized domains. NCQA enhanced transparency by integrating measurable quality indicators into healthcare plans, while URAC defined the trajectory of healthcare accreditation in the digital era. Collectively, these five organizations sequentially shaped the institutional, sectoral, and technological evolution of healthcare accreditation.

When the historical development of healthcare accreditation is analyzed along three main axes, the evolution of quality approaches and the complementary contributions of institutions become more evident. These axes can be classified as classical quality, patient safety, and digitalization.

The classical quality axis focused on standardizing processes and institutionalizing quality management systems. Founded in 1947, ISO, with its quality management standards, provided indirect guidance not only in industrial production but also in healthcare services. The ISO 9000 series, in particular, laid the groundwork for process control, certification, and the application of management standards, thus embedding healthcare institutions' management and operational processes within a framework of quality assurance. The defining feature of this period was a quality approach that did not directly address patient safety but ensured systematic and traceable organizational functioning.

The patient safety axis formed the core of healthcare accreditation. Established in 1951, The Joint Commission (TJC), and in 1966, the Commission on Accreditation of Rehabilitation Facilities (CARF), were pioneering organizations in this regard. TJC institutionalized hospital accreditation by placing patient safety at the center and disseminated safety standards globally through surgical safety checklists, adverse event reporting, and clinical protocols. CARF institutionalized the patient safety approach in niche areas by focusing on rehabilitation, mental health, and services for individuals with disabilities. The common feature of the standards developed along this axis was the effort to safeguard patient safety through directly measurable indicators.

The digitalization axis gained momentum from the 1990s onward, with NCQA and URAC playing key roles. NCQA, through its HEDIS measurement set, made the quality of health plans traceable and comparable, thereby contributing to greater transparency in healthcare performance measurement. This approach laid the foundation for a data-driven perspective in health management. URAC, by developing accreditation programs for telehealth, e-health, and digital healthcare services, redefined quality and safety standards in the digital era. The defining feature of this period was the incorporation of new risks and opportunities introduced by digitalization into the institutional framework of healthcare quality.

Taken together, these three axes reveal the evolution of healthcare accreditation from a holistic perspective. ISO's classical quality approaches provided an institutional foundation for healthcare management processes; TJC and CARF, through the patient safety axis, integrated accreditation into clinical practice in a systematic manner; and NCQA and URAC, along the digitalization axis, transformed healthcare services into a new dimension. Thus, accreditation has evolved into a multidimensional process, not only involving the inspection of institutional structures but also integrating patient safety and technological transformation.

##### 3.2.4.6 Evaluation of themes and findings in relation to the three original contributions

###### 3.2.4.6.1 Accountability axis

Findings along the accountability axis indicate that accreditation is not a mere abstract claim of “institutional quality,” but rather a mechanism that explicitly names who bears responsibility. The review of mortality committees examining death cases in terms of quality and safety, laboratories assuming clear accountability for the timely reporting of critical values, the linking of medication management and errors to specific clinical and pharmaceutical roles, and the establishment of patient rights and ethics as a separate monitoring track—all demonstrate a clear allocation of responsibility within the institution for each category of risk. Recurrent themes such as *“The Role of the Mortality Committee in Quality and Safety,” “Laboratory Critical Value Reporting,” “Medication Errors,”* and *“Patient Rights and Ethics”* reveal that the task of monitoring safety and quality is not diffusely shared but assigned to actors who can be held accountable. This transforms accountability from a philosophical principle into a traceable practice of governance.

Moreover, this axis shows that accountability extends not only to clinical outcomes (e.g. *Acute Myocardial Infarction Treatment Processes and Outcomes*) but also to organizational behavior (e.g. *Staff Productivity and Motivation*, *Teamwork and Communication*, *Promotion of Patient Rights*). These themes suggest that quality monitoring is not confined to patient outcomes alone but encompasses the human behaviors and interdisciplinary coordination that produce those outcomes. Such a framework expands the accountability mechanism to include clinicians, administrators, educators, and patient rights advocates under a common governance structure—thus reframing safety as a matter not merely of reporting results but of carrying role-based responsibility.

###### 3.2.4.6.2 Operational bottlenecks axis

Findings under the operational bottlenecks axis concretely demonstrate that the quality improvement cycle breaks not at the system level but at very small operational decision points. The theme *“Hand Washing Compliance Rates”* indicates that hand hygiene adherence still requires dedicated monitoring, highlighting where infection control collapses—not on paper, but through lapses in field behavior. Similarly, *“Patient Handover Systems”* identifies the complete and standardized transfer of information during patient transitions as a critical node, revealing that continuity of care often breaks down not at the protocol level but in communication during shift changes. These findings reframe the quality problem from a macro-level outcome such as “high hospital infection rates” to a micro-level process question: “At what moment, and between which individuals, did information fail to transfer?”

Within the same axis, the themes *“Discharge Instructions Affecting Re-hospitalization of Patients”* and *“Factors Affecting Re-hospitalization of Patients”* show that the quality of discharge instructions constitutes an independent breakpoint influencing readmission risk. Readmission is therefore not explained solely by clinical severity or socioeconomic status; rather, the adequacy of guidance, education, and follow-up planning at discharge emerges as a standalone risk line. This indicates that the improvement cycle must extend beyond inpatient care to include post-discharge follow-up; otherwise, quality indicators remain confined to the acute care phase. In other words, the bottleneck does not appear inside the clinic but at the moment the patient leaves it—and accreditation frameworks now define this transition as a measurable quality problem.

###### 3.2.4.6.3 Role architecture axis

Findings on the role architecture axis demonstrate that regulatory expectations are increasingly organized around information management, digital traceability, and technology-assisted care. However, these expectations do not naturally align with a single professional role. The simultaneous emergence of themes such as *“Information Technology and Electronic Health Record,” “Order Sets for Clinical Decision Support,” “Telemedicine and Remote Health Services,”* and *“Radiation Safety and Management”* suggests that accreditation logic now encompasses not only compliance with clinical protocols but also the embedding of decision-support systems, the safety of remote care processes, and the traceability of radiation dose management. Each of these areas brings with it overlapping layers of responsibility—clinical (appropriate treatment and dosage), technical (system setup and standardization of decision-support sets), and managerial (operation of telemedicine services in line with regulatory and quality standards).

This constellation reframes the institution's role architecture in the following way: regulators no longer merely state “use technology,” but rather mandate “manage patient safety, data integrity, radiation safety, and clinical continuity together while using technology.” Yet, in practice, this integrated expectation often remains fragmented: IT units handle technical integration, clinicians manage the implementation of decision-support systems, quality departments oversee traceability, while radiology and oncology services ensure dose safety. The dispersion of the same standard across multiple vulnerable points transforms noncompliance from a question of “what percentage of the standard is met” into one of “who owns this standard institutionally.” By making this fragmentation visible, the findings frame the alignment of technology-based accreditation expectations with clear, traceable organizational ownership as a *governance problem*—one that lies at the core of the institutional role architecture debate.

## 4. Discussion and limitations

This article aims to examine the role of accreditation organizations (AACs) that contribute to the accreditation process in health services through a bibliometric analysis. These organizations, which are effective in quality and patient safety in healthcare, have been discussed in large number of literature (Roberts *et al*., 1987; Griffith *et al*., 2002; Chen *et al*., 2003; Greenfield and Braithwaite, 2008; El-Jardali *et al*., 2008; Donaldson *et al*., 2000; Meinberg and Stern, 2003; Haynes *et al*., 2009; Donahue and Vanostenberg, 2000; Needleman *et al*., 2002). However, there is a lack of studies that comprehensively address this literature.

It is widely recognized that accreditation bodies have a critical role in healthcare. First, they continuously monitor and evaluate the practices of healthcare providers by establishing basic quality standards (Roberts *et al*., 1987; Griffith *et al*., 2002; Chen *et al*., 2003). They primarily focus on critical issues in terms of patient safety, such as preventing clinical errors and improving patient outcomes (Haynes *et al*., 2009).

As a result of the literature review, it has been observed that studies related to ABIH focus on various themes. For example, themes such as prevention of clinical errors, hospital quality, and theoretical foundations are noteworthy (Roberts *et al*., 1987; Griffith *et al*., 2002; Chen *et al*., 2003; Donaldson *et al*., 2000; Meinberg and Stern, 2003; Haynes *et al*., 2009). This diversity can be challenging for health services management and policymakers, as the existing literature addresses many different themes and methodologies without providing a coherent framework.

The accreditation process of healthcare organizations can positively impact important areas such as patient safety, efficiency and effectiveness. Accreditation can contribute positively to effectiveness factors such as promoting evidence-based medical practices and increasing the use of clinical indicators. It can also increase employee satisfaction and teamwork, leading to a more coordinated patient care process. However, this process may also bring disadvantages, such as financial burden and management complexity (Lewis and Hinchcliff, 2022). Therefore, it is necessary to consider the potential disadvantages and advantages of accreditation.

When we evaluate our findings in terms of co-citation analyses, we conclude that the importance and impact of accreditation processes require a multidimensional analysis. This issue is addressed under various themes in the literature. In this context, examining the reflections of different thematic focuses on accreditation is useful. The theme “Prevention of Clinical Errors and Patient Safety” emphasizes the critical role of accreditation bodies in protecting patient safety. This theme highlights the importance of accreditation processes that can provide a practical and institutionalized patient safety framework for healthcare. Furthermore, the theme “The Multifaceted Impact of Accreditation Processes on Healthcare Quality” implies that accreditation shapes healthcare quality through systemic and structural factors beyond individual procedures and services. This multifaceted impact shows that accreditation is not only a superficial quality control mechanism but also aims to improve the quality of healthcare services with a more holistic and inclusive approach.

The theme “Multidimensional Assessment of Hospital Quality in the United States,” which offers a US-centered perspective, can provide a basis for understanding how similar mechanisms work internationally. The contribution of this theme to the literature is to emphasize that hospital quality assessments should be considered not only from a local or national perspective but also from a global perspective. “Quality Assessment in Health Care: Theoretical Foundations and Personnel Management” focuses on the role of personnel management in balancing theoretical concepts with practical applications. In this context, accreditation processes aim to improve healthcare quality by bringing together theoretical and practical aspects.

From a historical viewpoint, Berwick's (1989) article explains how accreditation processes evolved. It helps trace their origins and shows how quality improvement strategies developed over time (Berwick, 1989). This article forms the foundations of modern approaches to accreditation processes. Furthermore, the theme “Accreditation and Health Policy in Pain Management” examines the impact of accreditation processes on specific health policies such as pain management and opioid use (Manchikanti *et al*., 2009, 2010; Phillips, 2000). Accreditation processes can improve quality in pain management and other areas by establishing specific quality standards.

An evaluation of co-occurrence analysis reveals multifaceted and complex accreditation-related relationships. For example, the words “safety” and “care” with high “Closeness” and “PageRank” values indicate issues that are central to the accreditation process. This shows how vital issues such as clinical safety and patient care are particularly emphasized by accreditation processes. Words with a high Betweenness value (e.g. “guidelines” and “risk”) act as a bridge between the different thematic clusters, indicating that the accreditation process is influential on a wide range of issues, from ethics to risk management.

Each thematic cluster focuses on different healthcare aspects of accreditation. For example, Cluster 3 (Patient Safety and System Failures) and Cluster 4 (Guidelines and Risk Management) reveal that accreditation in patient safety and risk management is essential. This finding illustrates how healthcare providers can use accreditation processes to improve patient safety and risk management. Furthermore, accreditation appears to address technical and clinical issues and more abstract issues such as ethics and beliefs (e.g. Cluster 2). The existence of these abstract issues emphasizes that accreditation is not only a technical requirement for healthcare providers but also involves ethical and social responsibilities.

The working themes of accreditation bodies reflect the evolutionary processes in the health sector and the needs that come to the fore in this process. According to the data obtained from the JCAHO publications examined in the themes of the first period, “Quality Indicators and Assessment”, “Drug Use and Management”, “Patient Safety”, “Procedure and Practice Improvements”, “Patient-Hospital Relationship” and “Emergencies and Risk Management” stand out. These themes show how much importance hospitals attach to the overall quality of healthcare services, medication management, patient safety, improvement of clinical procedures, relationships with patients, and emergency preparedness.

On the other hand, more specific topics such as “Quality and Performance Management”, “Hospital Operations and Management”, “Information and Technology Management” and “Patient Care and Treatment” have recently been among the themes emphasized by JCAHO for accreditation. This change reveals how technological developments in the health sector and the changing needs in hospital operations are reflected in the accreditation themes. In addition, the prominence of topics under the heading “Information and Technology Management” (e.g. Electronic Health Record, Telemedicine) emphasizes the importance of digitalization and technological advancement in the healthcare sector.

Overall, it is becoming clear that accreditation bodies have a multifaceted and complex role in healthcare. These organizations are effective in a wide spectrum, from preventing clinical errors to improving patient safety, from improving service quality to implementing risk management in the best way (Donaldson *et al*., 2000; Meinberg and Stern, 2003; Haynes *et al*., 2009; Lewis and Hinchcliff, 2022).

The finding of our study poses several challenges for healthcare managers and policymakers. While the existing literature addresses many different themes and methodologies, it does not provide a coherent framework. This can be a critical problem for decision-makers, especially in a complex and multidisciplinary field such as healthcare. It would be useful to conduct multidimensional analyses, as co-citation and co-occurrence analyses do, to better understand these various themes and the factors influencing accreditation processes. In particular, words with a high “Betweenness” value may be important to identify critical issues that can bridge different thematic areas.

The findings of this study carry multidimensional practical and strategic implications for healthcare managers, policymakers, and accreditation bodies. First, the thematic clusters and network analyses reveal both the research trends in the accreditation field and the central topics within knowledge networks, thereby concretely indicating the areas in which managers need to build capacity. For example, the central position of *patient safety* and *quality improvement* clusters in the literature demonstrates that these themes should be prioritized in the strategic plans of institutions (Braithwaite *et al*., 2017). From a policymaking perspective, the thematic gaps identified in this study highlight opportunities to develop policies in areas that have not yet been sufficiently explored. For instance, the limited number of studies on the impact of accreditation in certain geographic regions points to a pressing need for evidence-based policy in those contexts (Shaw, 2019). For accreditation bodies, the results offer a comparative perspective to evaluate both their own visibility and that of competing or complementary organizations in the literature. This enables institutions to make strategic decisions to strengthen their international visibility, enhance methodologies, and integrate more effectively into research networks.

Thematic clusters with high centrality and PageRank scores were identified as key knowledge domains.

These domains are likely to influence health policy and guide the development of accreditation standards. For example, the “Patient Safety Standards” cluster, consisting of highly cited studies, suggests that policymakers should reinforce national regulations on patient safety. Similarly, prominent metrics in the “Performance Measurement Systems” cluster can be adapted as KPI sets for use in healthcare managers' quality improvement strategies. Practical recommendations were also developed, such as integrating these findings into the training modules of accreditation bodies, enhancing data-driven evaluation criteria during site inspections, and prioritizing thematic focal points in national health plans. In this way, the study's outputs not only contribute to academic knowledge but also provide strategic roadmaps for policy and practice (Kapucu and Hu, 2020; Greenhalgh and Papoutsi, 2018).

This study diverges from recent bibliometric and systematic reviews along two main axes. First, in terms of scope, it adopts an actor-centered framework, shifting the focus away from accreditation domains themselves to the concrete contribution mechanisms generated by accreditation organizations within the process. Unlike model- or framework-focused reviews common in recent years, this approach makes organizational mechanisms—such as standard-setting, measurement and evaluation tools, evaluator and trainer capacity, feedback and benchmarking infrastructure—the primary units of analysis. This distinction separates “institutional contribution” from “general quality maturity” and prevents scope creep.

Second, methodologically, it deliberately aligns the bibliometric time series with the policy and standardization timeline. The 2005 peak observed in the publication–citation curve is not treated merely as a quantitative spike but as a concurrent inflection point where performance measurement and safety standards became institutionalized. The Hospital Quality Alliance's expansion of indicator sets for national public reporting in 2005, and the Joint Commission's core measures implemented since 2002, both reinforced the link between accreditation and performance evaluation. Thus, the analytical focus on the post-2005 sub-period aims to capture not only the bibliometric peak but also the simultaneous policy and standardization shift (Jha *et al*., 2005; Williams *et al*., 2005).

This dual-axis design provides a causal rationale for the often-overlooked question in recent reviews: *“Why this period?”* While publication growth or topic maps alone cannot explain structural turning points, this study strengthens interpretive power by anchoring periodization in policy-based milestones. As such, it offers a methodological alternative to approaches that justify period choices solely by data availability or trend visibility.

The thematic coding of contribution mechanisms also represents a distinctive innovation. It demonstrates how sub-mechanisms—such as standard development, metric and indicator architecture, training and evaluator pools, feedback and corrective action loops, external benchmarking, and transparent reporting—are interconnected. This framework positions organizations not merely as “accrediting bodies” but as institutional architects shaping measurement infrastructures and learning cycles. Whereas some recent reviews jump directly to outcome variables without elaborating these mechanical linkages, this study fills that gap.

The explicit requirement that inclusion and exclusion criteria directly test “institutional contribution” strengthens internal validity and sharpens the interpretive scope by excluding framework-heavy but actor-ambiguous studies. This choice does not wholesale exclude emerging trends but instead ensures selective coverage consistent with the research question; it also lays the groundwork for future reviews specifically targeting organizational contributions in digital and telehealth domains.

In conclusion, this study makes three concrete contributions beyond recent reviews: (1) adopting an actor-centered and mechanism-based scope; (2) integrating bibliometrics with the policy–standardization timeline; and (3) applying causal periodization with a focus on the post-2005 sub-period. This framework renders the role of accreditation bodies in the accreditation process visible not only through outcomes but also through the institutional tools and relational networks that make those outcomes possible, thereby adding distinct value to the literature (Jha *et al*., 2005; Williams *et al*., 2005).

The scope of this study is not the concept of accreditation *per se*, but the institutional contributions of accreditation bodies to the accreditation process. The unit of analysis is the organization-actor; contributions of bodies such as JCI, TJC, NCQA, and URAC in standard-setting, measurement and evaluation, training and evaluator development, feedback, and benchmarking mechanisms were thematically coded. For this reason, developments in 2020–2025 such as rapidly evolving digital health accreditation, telemedicine quality standards, and maturity/comparison models were excluded when they did not directly analyze the contributions of an accreditation body. The inclusion criterion of this study required that the research empirically address the concrete contribution mechanisms of an accreditation body to the accreditation process and report its findings through the organization's tools. A small number of records in the digital or telehealth domain met this condition and were reviewed, but studies not directly testing institutional contribution were excluded for reasons of consistency and internal validity. The rationales for exclusion were explicitly documented in the Methods section and visualized in Figure 1.

Finally, it is important to note that the themes that healthcare organizations and accreditation bodies work with the most have evolved over time and that this evolution aligns with the overall trends and needs in the healthcare sector. In this context, accreditation processes need to be continuously updated in line with the current challenges and needs faced by hospitals and other healthcare organizations. In short, the role of accreditation processes and organizations in healthcare is highly dynamic and multifaceted, with many opportunities and challenges that need to be managed effectively.

### 4.1 Societal impacts and policy implications

The societal implications of these findings manifest across three key axes. First, in the axis of *patient safety and clinical outcomes*, the widespread adoption of accreditation standards in core domains such as patient identification, medication management, and “never events” necessitates the strengthening of national regulations aimed at reducing mortality and morbidity. Second, in the axis of *equity and access*, the faster adoption of accreditation by institutions with stronger organizational capacity risks widening the quality gap between advantaged and disadvantaged regions; therefore, performance metrics should be balanced through regional weighting and targeted incentives. Third, in the *cost–transparency axis*, the institutionalization of performance measurement systems and reporting practices supports data-driven resource allocation and enhances public transparency. These three axes align directly with the study's highly central themes—*patient safety standards* and *performance measurement*—and may serve as key performance indicator (KPI) frameworks in national health policy plans.

The findings reveal that accreditation has evolved from being a technical instrument of hospital-level compliance into a governance tool with direct societal implications. Thematically central nodes identified through network analysis—such as *patient safety, clinical safety, medication management, pain management, reduction of readmissions, hand hygiene compliance, adequate discharge instructions, intra-team communication,* and *timely reporting of critical laboratory values*—demonstrate that accreditation is now tied to outcomes measurable at the population level, including mortality, readmission, infection spread, chronic disease control, and quality of life. Notably, the recurrence of themes like *“Patient Safety and Risk Management,” “Quality Indicators and Performance Measurement,” “Eradication of ‘Never Events’,” “Factors Affecting Re-hospitalization of Patients,”* and *“Patient Handover Systems”* across both highly cited and recent studies underscores this shift: accreditation increasingly addresses public outcomes such as the reduction of preventable harm, the continuity of post-discharge care, and the decrease of readmission rates—beyond mere institutional process standardization.

This trajectory implies two critical public health consequences. First, patient safety and quality improvement now extend beyond technical correctness at the bedside (e.g. procedural sedation safety, radiation safety) to encompass broader axes of inequality. Second, long-term determinants such as the quality of post-discharge care and *health literacy* (e.g. *“Health Literacy and Patient Education”*) have become legitimate domains of accreditation. Accordingly, the study demonstrates how accreditation extends its reach beyond institutional compliance to encompass social cost domains such as hospital readmission burden, opioid and pain management policies, infectious risk control, chronic disease management, and end-of-life care quality. This framing suggests that accreditation can serve as a governance mechanism for equity, transparency, and public accountability within national health systems.

Furthermore, the findings show that accreditation themes are not merely academic constructs but can be operationalized into directly manageable capacity areas. Themes such as *“Quality Indicators and Performance Measurement,” “Teamwork and Communication,” “Staff Productivity and Motivation,” “Hospital Operations and Management,” “Information Technology and Electronic Health Record,” “Order Sets for Clinical Decision Support,” “Telemedicine and Remote Health Services,” “Radiation Safety and Management,”* and *“Audit according to Accreditation Standards for Emergencies”* collectively outline explicit operational modules for hospital administrators.

This list yields three actionable managerial imperatives. First, the *selection of priority capacity domains* to be included in institutional strategic plans: if patient safety and quality improvement clusters occupy central positions in the network, they should be defined as distinct project lines within the quality program and monitored through KPI sets (e.g. readmission rate, critical value reporting time, hand hygiene compliance)—a prioritization made concrete by this study. Second, the *closure of the gap between clinical care and managerial infrastructure*: the simultaneous rise of themes such as electronic health records, standardized order sets for decision support, safe telemedicine operation, radiation dose tracking, and emergency preparedness reveals the need to treat quality not only as a clinical protocol issue but as one intrinsically linked to information systems and operational management. Third, the *role architecture and accountability schema*: themes such as mortality committee oversight, critical laboratory value reporting, standardization of discharge instructions, intra-team communication, and patient rights advocacy are distributed across different professional groups. By making this distribution visible, the study renders administratively answerable the question of “who is responsible for which standard, and how should each feedback loop be closed?”—thereby enabling the direct translation of accreditation findings into an internal process ownership matrix and producing a practically applicable governance tool.

Linking with Theoretical Framework.

The findings obtained in this study can be associated with two theoretical approaches commonly used to explain quality and accreditation in healthcare services: Donabedian's Quality Model and the theory of Neo-Institutionalism.

Donabedian's Quality Model provides a holistic framework for evaluating healthcare quality through structural, process, and outcome dimensions (Donabedian, 1988a, b). Themes emerging from our study, such as *“error prevention and patient safety,” “multidimensional quality measurement,”* and *“organizational cost-performance dynamics”* directly align with the process and outcome dimensions of this model. The standard-setting, auditing, and feedback mechanisms of accreditation bodies strengthen the structural dimension of the model. In this sense, accreditation processes play not only an evaluative role but also a decisive function across all quality layers defined by Donabedian.

The neo-institutionalist perspective, on the other hand, explains accreditation not merely as a technical tool of quality management but also as a mechanism that provides organizational legitimacy. As emphasized by Meyer and Rowan (1977) and DiMaggio and Powell (1983), organizations adopt specific structures and processes not only for efficiency and effectiveness but also to gain institutional legitimacy. The findings highlighted in our study, such as *“international standards,” “comparative benchmarking,”* and *“institutional collaborations,”* demonstrate that healthcare organizations participate in institutional isomorphism processes through accreditation. This enables healthcare organizations to consolidate their legitimacy by aligning with similar standards at both local and global levels.

In conclusion, Donabedian's Quality Model explains how accreditation links to the structure, process, and outcomes of healthcare services. Neo-institutional theory complements this view by showing how these processes become institutionalized and create legitimacy within organizations. Taken together, these two approaches provide a comprehensive understanding of accreditation as both a technical quality outcome and a determinant of organizational behavior and policy.

The societal implications of these findings materialize along three distinct axes.

First, the axis of patient safety and clinical outcomes: the widespread adoption of accreditation standards in core domains such as patient identification, medication management, and *“never events”* necessitates the strengthening of national regulations aimed at reducing mortality and morbidity.

Second, the axis of equity and access: the faster adoption of accreditation by institutions with stronger organizational capacity may widen the quality gap between advantaged and disadvantaged regions; therefore, performance metrics should be balanced through regional weighting and targeted incentive mechanisms.

Third, the axis of cost and transparency: the institutionalization of performance measurement systems and reporting practices facilitates data-driven resource allocation and enhances public transparency.

Together, these three axes align directly with the study's highly central themes—*patient safety standards* and *performance measurement*—and can be translated into measurable indicators usable as Key Performance Indicator (KPI) sets in national health policy planning.

## 5. Limitations

This study has several limitations. First, due to the inherent nature of bibliometric analyses, the dataset used here does not represent a randomly selected sample. It is composed of publications retrieved from the *Web of Science (WoS) Core Collection* database using predefined keywords, restricted to specific document types and language (English only). Consequently, the results give greater weight to the English-language literature and to journals indexed by WoS. Furthermore, because WoS does not systematically cover records prior to 1975, early-period studies, local or national journals, and non-English contributions are not fully reflected (Fuat *et al*., 2023; Arbak *et al*., 2025). Therefore, the generalizability of the findings is limited—not to the entire historical and geographical production of the field, but rather to the *visible core* encompassed by this particular search strategy.

Second, in conducting the bibliometric network analyses (citation, co-citation, co-occurrence, and co-authorship), we employed the *default parameters* of the *bibliometrix* package and the commonly accepted threshold values used in the literature. For instance, in generating keyword co-occurrence and reference co-citation networks, setting a minimum frequency or threshold value may lead to the exclusion of low-frequency but potentially *emerging themes*. Conversely, lowering the threshold excessively would result in fragmented, non-interpretable, and analytically weak clusters. Thus, the selected thresholds were chosen to yield interpretable cluster structures; however, this choice inevitably influences the analysis sensitivity—affecting which nodes are positioned at the center and which are pushed to the periphery of the network.

Finally, these methodological characteristics do not allow for causal inference. Network structures indicate *relational proximity, clustering,* and *co-occurrence* patterns, but they do not establish directional causality. These limitations highlight two key directions for future research: (1) incorporating *non-WoS data sources* such as Scopus, regional indexes, and non-English journals, and (2) performing *sensitivity analyses* to test the robustness of threshold parameters used in network generation. Such expansions would help make *non-English knowledge production* and *early historical contributions* more visible within the global academic landscape (Fuat *et al*., 2023; Cinoğlu and Kurutkan, 2021).

## 6. Conclusion

In conclusion, the accreditation process and accreditation bodies (ABIH) role in health services are complex and multidimensional. This study has explored in depth the different themes focused on in the literature and how these issues are reflected in accreditation processes. In particular, a framework has been developed that combines themes such as patient safety, quality assessment, theoretical foundations, and personnel management. Co-citation and co-occurrence analyses reveal how these themes function in the accreditation process and how they contribute to health services. In this context, thematic clusters focusing on critical issues such as patient safety and risk management show that accreditation is vital for healthcare providers. In addition to technical and clinical issues, more abstract issues such as ethics and beliefs were also identified as being involved in the accreditation process.

The themes that accreditation bodies work with the most have evolved over time and reflect the changing needs of healthcare. This emphasizes that accreditation processes are dynamic and constantly being updated. As a result, it was concluded that accreditation processes can positively impact critical areas such as effectiveness, efficiency, and especially patient safety in healthcare services. There may also be possible disadvantages such as financial burden and management complexity. This study can be an important reference for health management and policymakers to understand the complexity of accreditation processes and to manage these processes more effectively.

This study demonstrates that accreditation themes are not merely technical compliance items but governance instruments that redefine societal outcomes, capacity priorities, and the architecture of accountability.

## Data Availability

The data and materials used in this study are available upon request.
